# Beyond the Status Quo: A Role for Beta Oscillations in Endogenous Content (Re)Activation

**DOI:** 10.1523/ENEURO.0170-17.2017

**Published:** 2017-08-02

**Authors:** Bernhard Spitzer, Saskia Haegens

**Affiliations:** 1 Department of Experimental Psychology, University of Oxford, Oxford OX1 3UD, United Kingdom; 2 Department of Neurological Surgery, Columbia University College of Physicians and Surgeons, New York, NY 10032; 3 Centre for Cognitive Neuroimaging, Donders Institute for Brain, Cognition and Behaviour, Radboud University Nijmegen, Nijmegen 6500 HB, The Netherlands

**Keywords:** beta rhythm, decision making, network interactions, neural oscillations, top-down control, working memory

## Abstract

Among the rhythms of the brain, oscillations in the beta frequency range (∼13–30 Hz) have been considered the most enigmatic. Traditionally associated with sensorimotor functions, beta oscillations have recently become more broadly implicated in top-down processing, long-range communication, and preservation of the current brain state. Here, we extend and refine these views based on accumulating new findings of content-specific beta-synchronization during endogenous information processing in working memory (WM) and decision making. We characterize such content-specific beta activity as short-lived, flexible network dynamics supporting the endogenous (re)activation of cortical representations. Specifically, we suggest that beta-mediated ensemble formation within and between cortical areas may awake, rather than merely preserve, an endogenous cognitive set in the service of current task demands. This proposal accommodates key aspects of content-specific beta modulations in monkeys and humans, integrates with timely computational models, and outlines a functional role for beta that fits its transient temporal characteristics.

## Significance Statement

Brain oscillations at frequencies of 13–30 Hz (the beta rhythm) are traditionally associated with sensory and motor processing, but are increasingly implicated in various cognitive functions, such as working memory (WM) and decision making. Here, we review new evidence that beta activity in these domains can be content specific, that is, it can reflect the very information that is currently being processed. Going beyond previous accounts that link beta to maintenance of the current brain state, our review highlights the dynamic, often short-lived nature of beta modulations during endogenous information processing. We integrate these findings in a dynamic network view where beta-synchronization supports the internally driven (re)activation of neuronal ensembles to represent currently task-relevant contents.

## Introduction

### Beta-band oscillations: beyond motor control

Oscillations in the beta frequency range (∼13–30 Hz) have traditionally been associated with sensorimotor processing (Hari and Salmelin, 1997; Pfurtscheller and Lopes da Silva, 1999). During preparation and execution of movements, beta oscillations in sensorimotor cortex show marked power decreases (assumed to reflect local desynchronization), followed by a “rebound” of power (i.e., synchronization) after movement (Kilavik et al., 2013). A similar sequence of beta power changes is observed in somatosensation, with desynchronization in anticipation of and during stimulation, followed by (re)synchronization after stimulus offset (Bauer et al., 2006; Spitzer et al., 2010; Van Ede et al., 2010). The dynamics of beta activity in sensorimotor cortex often resemble and/or parallel modulations of alpha band activity (∼8–12 Hz), in that power decreases with active engagement, for instance during spatial attention (Bauer et al., 2006; Schubert et al., 2009; Jones et al., 2010; Van Ede et al., 2011; but see Haegens et al., 2012). However, whereas alpha oscillations are widely linked to the inhibition of task-irrelevant areas (Klimesch et al., 2007; Jensen and Mazaheri, 2010; Haegens et al., 2011a), the precise functional role of sensorimotor beta-synchrony remains unclear. Initially believed to reflect cortical idling (Pfurtscheller et al., 1996; Neuper and Pfurtscheller, 2001), more recent views suggest a role in maintaining the current sensorimotor set, or “status quo” (Engel and Fries, 2010; cf. Jenkinson and Brown, 2011).

**Figure 1.**
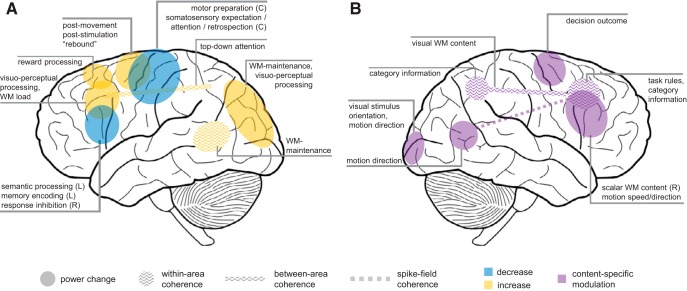
Beyond motor control. Schematic overview of oscillatory beta-band effects across cortex. ***A***, Overall beta activity changes (in-/decreases) associated with different cognitive functions (NB, nonexhaustive schematic). ***B***, Content-specific modulations of beta-band activity; see text for details. For convenience, findings from human- and nonhuman primate studies are illustrated on a common template. Unless specified (L, left; R, right; C, contralateral), effects were not systematically lateralized.

Beyond its established role as a sensorimotor rhythm, beta activity has been observed in various different cortical areas and is increasingly implicated in a wider range of cognitive functions (Engel and Fries, 2010). Modulations of beta oscillatory activity in nonsomatomotor areas (e.g., frontal, parietal, visual; Fig. 1*A*) have been associated with visual perception (Donner et al., 2007; Piantoni et al., 2010; Kloosterman et al., 2015), language processing (for review, see Weiss and Mueller, 2012), working memory (WM; Tallon-Baudry et al., 1998; Deiber et al., 2007; Axmacher et al., 2008; Siegel et al., 2009), long-term memory encoding and retrieval (Sederberg et al., 2006; Hanslmayr et al., 2009; Spitzer et al., 2009; for review, see Hanslmayr et al., 2016), decision making (Pesaran et al., 2008; Wimmer et al., 2016; Wong et al., 2016), response inhibition (Jha et al., 2015), and reward processing (for review, see Marco-Pallarés et al., 2015). In some of these contexts, beta-band modulations occur in a relatively low frequency band (“lower” beta, ∼13–20 Hz) and in tandem with alpha (Hanslmayr et al., 2009). In other cases, beta-band rhythms of varying frequencies (including “upper” beta, ∼20–30 Hz) behave in ways more similar to gamma activity (>30 Hz) and increase, rather than decrease, with task-related engagement (Tallon-Baudry et al., 1998; Marco-Pallarés et al., 2015; Kornblith et al., 2016).

While a unifying theoretical account of cortical beta oscillations is currently lacking, some mechanistic aspects have been tentatively identified. In particular, beta oscillations are mostly associated with endogenous, top-down-controlled processing (Buschman and Miller, 2007; for review, see Engel and Fries, 2010; Wang, 2010; Fries, 2015). Furthermore, in line with a “communication through coherence” view (Fries, 2005, 2015), oscillations in the beta frequency range are assumed to facilitate long-range interactions on a cortical network level (Kopell et al., 2000; Varela et al., 2001; Benchenane et al., 2011; Kilavik et al., 2013). Both these aspects have been integrated in a predictive coding framework, where gamma-synchronization serves feedforward (bottom-up) communication, whereas beta-synchronization affords feedback communication of top-down predictions (Arnal and Giraud, 2012; Bastos et al., 2012; Bastos et al., 2015; Michalareas et al., 2016). One of the many persistent puzzles regarding beta, however, remains its unclear relation to neuronal activity as measured in spike firing rates and/or blood-oxygen-level dependent (BOLD) signals. Whereas oscillations in other frequency bands are known to correlate either positively (e.g., gamma) or negatively (e.g., alpha) with these activity measures, findings for beta have been mixed (e.g., Michels et al., 2010; Hanslmayr et al., 2011), with some studies showing no correlation at all (e.g., Whittingstall and Logothetis, 2009; Rule et al., 2017).

Perhaps surprisingly in light of the above, an increasing number of findings indicate that in some task contexts, beta oscillatory activity can be content specific, that is, it can reflect the very information that is currently being processed (Fig. 1*B*). Across human and monkey species, content-specific beta activity was found to carry information about internalized task rules (Buschman et al., 2012), stimulus categories (Antzoulatos and Miller, 2014, 2016; Stanley et al., 2016), scalar magnitudes (Spitzer et al., 2010; Spitzer and Blankenburg, 2011; Spitzer et al., 2014a) and other stimulus properties (Salazar et al., 2012; Mendoza-Halliday et al., 2014; Lewis et al., 2016; Wimmer et al., 2016), as well as subjective comparison outcomes (Haegens et al., 2011b; Herding et al., 2016). Such content-specific beta activity has in particular been observed during endogenous information processing in WM and decision making. Before considering these two domains in greater detail, we briefly discuss by which neurophysiological mechanisms beta oscillations might be generated.

### Generation of cortical beta oscillations

Two main views exist in the literature, suggesting that (1) beta is generated in cortex (Jensen et al., 2005; Roopun et al., 2006; Kramer et al., 2008; Kopell et al., 2011; Sherman et al., 2016), or (2) that beta is generated in the basal ganglia and propagated to cortex via the thalamus (Holgado et al., 2010; McCarthy et al., 2011). Within the view of cortically generated beta, one class of models suggests that beta is generated by local spiking interactions among cells, either consisting of pyramidal cell-interneuron loops (Jensen et al., 2005; Kramer et al., 2008; Kopell et al., 2011; Lee et al., 2013) or layer 5 pyramidal cells coupled via gap junctions (Roopun et al., 2006). A more recent proposal (Sherman et al., 2016) suggests an intermediate model, with beta being generated in cortex but depending on a (laminae-specific) exogenous drive originating from subcortical and/or cortical influences (see also Schmiedt et al., 2014, suggesting cortical beta generation driven by thalamic and/or top-down cortical inputs).

Based on local generator models, it has been suggested that beta is ideally suited for flexibly and dynamically forming cell assemblies (Roopun et al., 2008; Kopell et al., 2011), and for long-distance inter-area communication (Kopell et al., 2000). These models rely on local spiking interactions between excitatory and inhibitory neurons, and on intrinsic currents of the underlying pyramidal cells (e.g., h-currents or m-currents, determining the cell’s rebound after hyperpolarization), defining the time constants for spike firing, thereby contributing to beta rhythmogenesis (Roopun et al., 2008; Kopell et al., 2011). Kopell et al. (2011) suggest that beta-synchronized cell assemblies are robust as they are self-sustaining after a long, decaying excitatory input [contrary to pyramidal-interneuron gamma (PING)-based networks, which need ongoing input; PING, a model of local circuit gamma generation], and can concurrently exist with other cell assemblies (again, contrary to PING assemblies which compete with one another as they rely on the same inhibitory interneurons). The sustained nature of these cell assemblies, spiking at a low beta rate (∼15 Hz), would allow maintaining of neuronal activity patterns, i.e., a mechanism for WM, and the linking of past and present inputs.

More recently, Sherman et al. (2016) proposed a model building on prior work (Jones et al., 2007; Jones et al., 2009; Sacchet et al., 2015), where cortical beta is generated in the apical dendrites of large populations of spatially aligned pyramidal neurons, which span several layers. Specifically, this model produces transient beta activity (<150 ms) by the integration of simultaneous (subthreshold) excitatory drives to the proximal (closer to the soma) and distal apical dendrites of pyramidal cells located in supragranular (layers 2/3) and infragranular layers (layer 5). The weaker proximal drive (of ∼100-ms duration) arrives via granular (layer 4), the stronger distal drive (∼50 ms) via the supragranular layers, with both extrinsic drives arising from thalamic or potentially higher-order cortical areas. The model accurately generates beta “burst” events (<150 ms), with a nonsinusoidal wave form as observed in spontaneous human, monkey and rodent recordings (Sherman et al., 2016). Moreover, within this framework, when both drives arrive nearly synchronously at a 10-Hz rate, a sustained beta rhythm can be produced. Note that a beta-rate input is neither required nor sufficient for this model to produce realistic beta events (contrary to models that assume generators in the basal ganglia), nor do individual cells fire at a beta rate (in contrast with other local generator models). Rather, beta oscillatory activity arises from net subthreshold dendritic fluctuations, relying on integration of feedforward (to granular layer) and, critically, feedback inputs (to supragranular layers).

## Beta-band oscillations in WM

Numerous studies have reported beta power increases, concomitant with modulations in other frequency bands, during WM maintenance of visual (Tallon-Baudry et al., 1998; Liebe et al., 2012; Lara and Wallis, 2014; Wimmer et al., 2016), verbal (Deiber et al., 2007), or temporal information (Chen and Huang, 2016). Such effects occur in frontal, parietal, and/or temporal areas, and can vary with WM load, i.e., the amount of to-be-maintained information (Deiber et al., 2007; Honkanen et al., 2015; Chen and Huang, 2016; see also Palva et al., 2011; Kornblith et al., 2016). In addition, several studies have shown that WM demands can alter the degree to which beta oscillations are phase synchronized, both within and between cortical areas (Tallon-Baudry et al., 2001; Babiloni et al., 2004; Tallon-Baudry et al., 2004; Axmacher et al., 2008; Salazar et al., 2012; Dotson et al., 2014).

Findings of enhanced beta activity in WM tasks appear consistent with a role in actively maintaining the current cognitive set, i.e., the status quo (Engel and Fries, 2010). However, overall changes of oscillatory activity during WM processing can depend on various task factors and are also often found in frequency bands other than beta, especially theta (4–7 Hz), alpha, and gamma (for review, see Fell and Axmacher, 2011; Roux and Uhlhaas, 2014). While overall activity changes may reflect involvement in WM, more direct insights into the mechanisms of WM storage can be gained from delay activity that reflects the current memory content, in terms of the task-relevant stimulus information that is to-be-maintained on a given trial (Christophel et al., 2017). Such content-specific delay activity has traditionally been observed in persistent neuronal spiking (e.g., Kubota et al., 1974; Miller et al., 1996), local gamma-band activity (Pesaran et al., 2002), as well as BOLD activity patterns (Harrison and Tong, 2009). However, a growing body of recent literature indicates that WM contents can also be reflected in oscillatory brain signals, particularly in the beta frequency range (Spitzer et al., 2010; Spitzer and Blankenburg, 2011; Salazar et al., 2012; Mendoza-Halliday et al., 2014; Antzoulatos and Miller, 2016; Rose et al., 2016; Wimmer et al., 2016).

### Content-specific modulations in scalar WM

**Figure 2.**
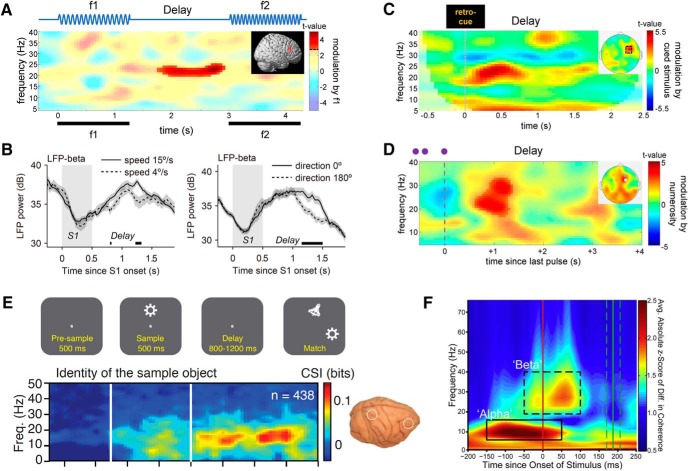
Content-specific beta activity during WM processing. ***A***, During WM maintenance of vibrotactile frequency information, prefrontal EEG beta power is parametrically modulated by the frequency of the to-be-maintained stimulus (f1). Adapted with permission from Spitzer et al. (2010). ***B***, Similar beta power modulations were found in LFP recordings in monkey PFC during WM maintenance of visual motion information. Adapted with permission from Wimmer et al. (2016). Note that in standard delay tasks, beta modulations occurred mostly late in the WM delay period. ***C***, In contrast, when participants were retro-cued to focus on a given WM content for further maintenance, beta was modulated early after the cue. Adapted with permission from Spitzer and Blankenburg (2011). ***D***, Similar transient modulations were observed during WM processing of the approximate number of previously presented inputs (three to eight pulses in rapid sequence, illustrated in purple). Adapted with permission from Spitzer et al. (2014a). ***E***, Content-specific fronto-parietal beta-synchronization during WM maintenance of visual object information in monkeys. As in ***A–D***, these effects were absent during stimulus encoding. Adapted with permission from Salazar et al. (2012). Reprinted with permission from AAAS. ***F***, Rule-dependent beta-synchronization in monkey PFC at the time of the to-be-evaluated stimulus (see also Fig. 4*C*). Adapted with permission from Buschman et al. (2012). Reprinted with permission from Elsevier.

One line of evidence for content-specific delay activity in the beta-band comes from studies of WM for scalar magnitudes, such as the speed, intensity, or duration of a stimulus. The neural basis of scalar information processing has been studied in great detail in a classic somatosensory task (Mountcastle et al., 1967; Romo and de Lafuente, 2013), where monkeys were trained to memorize the frequency of a brief tactile vibration (f1) for delayed comparison against a second vibration (f2; Fig. 2*A*, top). As a seminal finding in this task, the trial-specific f1 frequency is encoded parametrically, i.e., in a monotonically graded fashion, in neuronal firing rates throughout the cortical processing hierarchy (Romo and de Lafuente, 2013). During the WM delay after f1, such parametric coding prevails in prefrontal cortex (PFC) and premotor cortex, with different cell populations either positively or negatively tuned to the frequency of f1 (Romo et al., 1999; Hernández et al., 2002; Barak et al., 2010). In subsequent human EEG experiments, similar effects were observed in prefrontal beta activity, with parametric modulations of oscillatory power as a function of f1 frequency (Spitzer et al., 2010; Spitzer and Blankenburg, 2011; Ludwig et al., 2016). Further studies showed that such beta power modulations are not specific to vibrotactile frequency information, but can also be observed for other analog continua, like stimulus intensity, motion speed, or approximate number (Spitzer et al., 2014a,b; Wimmer et al., 2016). A general picture emerging from this line of work is that at least some of the computations underlying scalar WM are supramodal (Spitzer and Blankenburg, 2012; Vergara et al., 2016; see also Nieder, 2012), potentially reflecting high-level abstractions of the task-relevant magnitude, rather than concrete sensory information (Spitzer et al., 2014a,b; see also Christophel et al., 2017).

### Content-specific (de)synchronization within and across areas

The modulations of beta activity in scalar WM tasks were observed in power measures, which are thought to reflect the local (de)synchronization of neuronal ensembles within a brain area (Pfurtscheller and Lopes da Silva, 1999). However, using more complex stimuli, content-specific WM activity in the beta-band has also been observed on a larger-scale cortical network level. For instance, in simultaneous recordings from prefrontal and parietal cortex in behaving monkeys, information about memorized visual objects could be decoded not only from sustained spiking in either region, but also from the level of beta-band synchronization between regions (Salazar et al., 2012) (Fig. 2*E*). Similarly, beta-synchronization of prefrontal networks was found to reflect currently relevant task-rules (Buschman et al., 2012) (Fig. 2*F*, 4*C*) and stimulus categories (Antzoulatos and Miller, 2014, 2016; Stanley et al., 2016). In another recent study, memory-coding neurons in monkey lateral PFC were found to be synchronized via beta oscillations to motion-sensitive area MT, in which the memorized motion information was also reflected in local field potential (LFP) power but not in spiking activity (Mendoza-Halliday et al., 2014). The latter findings were interpreted as evidence for top-down signaling from PFC to lower-level visual areas, in line with the emerging notion of beta oscillations as a communication channel for top-down and feedback information across cortex (Buschman and Miller, 2007; Bastos et al., 2015).

### A role in (re)activating WM content

Several lines of evidence suggest that content-specific beta oscillations do not reflect a persistent memory trace per se, but rather, a class of endogenous processes that are auxiliary to WM processing. For instance, content-dependent beta activity is typically not sustained throughout entire memory delays (of several seconds), but relatively short lived, often lasting only several hundreds of milliseconds (Spitzer and Blankenburg, 2011; Salazar et al., 2012). Furthermore, unlike WM codes in neuronal spiking (Romo et al., 1999; Barak et al., 2010), prefrontal beta modulations are usually absent during stimulation, and seem to emerge only at particular times during WM retention (Fig. 2*A-D*). More specifically, in standard delay tasks, beta modulations are mostly found late in the delay (Spitzer et al., 2010; Salazar et al., 2012; Spitzer and Blankenburg, 2012; Wimmer et al., 2016), when WM information might be endogenously (re)activated or “refreshed” (Johnson, 1992) in preparation for the imminent comparison task (Spitzer et al., 2010; Myers et al., 2015; Wimmer et al., 2016). In contrast, when participants were explicitly (retro)-cued to update WM with one of two previously presented stimuli for further maintenance, prefrontal beta was modulated early after the cue, and therein selectively reflected the cued stimulus information (Spitzer and Blankenburg, 2011; Spitzer et al., 2014b). Similarly, in tasks where the scalar magnitude of a stimulus could only be assessed after integration over time (e.g., the number of pulses in a sequence), beta modulations occurred promptly after the accumulation period, as if the task-relevant scalar was “activated” in WM as soon as it was internally computed (Spitzer et al., 2014a,b). Taken together, rather than a substrate of persistent memory storage per se, prefrontal beta modulations seem to reflect the momentary updating, or (re)activation, of WM content in the service of the task at hand.

To our knowledge, content-specific beta activity in WM has thus far only been observed during single-item maintenance (Salazar et al., 2012; Dotson et al., 2014; Mendoza-Halliday et al., 2014), and several findings suggest that the capacity of beta-associated WM updating might be limited to a single piece of information in the current focus of attention (Spitzer and Blankenburg, 2011; Spitzer et al., 2014b; Antzoulatos and Miller, 2016; Wimmer et al., 2016; cf. Oberauer, 2002). However, one recent study showed that an additional, currently unattended memory item can be pushed into an active WM state by transcranial magnetic stimulation (TMS) of WM-coding areas (Rose et al., 2016). Interestingly, whereas the currently attended memory information could be decoded from various EEG frequency bands, the TMS-induced reactivation of the unattended memory item was exclusively evident in content-specific beta activity. In other words, beta activity specifically marked the transition of “latent” WM contents (see below; Mongillo et al., 2008; Stokes, 2015) into an active memory, consistent with a role of beta in updating, or reactivating, information in the current focus of WM.

### Neurocomputational perspectives on beta oscillations in WM

A role of beta oscillations in WM has also been put forward in computational modeling work (Kopell et al., 2011; Lundqvist et al., 2011; Dipoppa and Gutkin, 2013). Simulations by Kopell et al. (2011), for instance, showed beta oscillations to be uniquely suited to form and coordinate cell assemblies for sustained stimulus processing in the absence of further input, eventually permitting the coexistence of past and present stimulus information in the same network. In this view, beta rhythms may scaffold functional assemblies for active WM processing. Another line of modeling studies, with a focus on multi-item WM, suggests a sequential replay of individual WM items (see also Lisman and Jensen, 2013), in terms of alternations between “ground” and “active” states (see below), where the former is dominated by alpha/beta and the latter by gamma (Lundqvist et al., 2010; Lundqvist et al., 2011). Corroborating this idea, Lundqvist et al. (2016) reported a dissociation between beta and gamma during multi-item WM in monkey PFC, where gamma bursts were associated with stimulus encoding and decoding in spikes, whereas beta bursts prevailed during memory maintenance. However, it was not analyzed whether beta and/or gamma bursts themselves carried information about the WM contents, leaving the question of content-specific beta activity in multi-item WM to future research (but see Siegel et al., 2009, for a potential role of beta phase in multi-item WM).

## Beta-band oscillations in decision making

Given the well-documented involvement of beta-band oscillations in movement preparation (Murthy and Fetz, 1992; Sanes and Donoghue, 1993; Crone et al., 1998; Pfurtscheller and Lopes da Silva, 1999), it seems not surprising that sensorimotor beta effects are routinely observed in decision-making tasks where choices are to be communicated via a motor response (Kaiser et al., 2007; Zhang et al., 2008; Bidet-Caulet et al., 2012). During perceptual discrimination of auditory stimuli, for instance, the latency of preparatory beta power modulation was found to mimic response time differences across varying levels of task difficulty (Kaiser et al., 2007). Such effects typically manifest as sensorimotor power decreases contralateral to the to-be-moved limb, and are commonly assessed using lateralization indices (e.g., contrasting left- vs. right-hemispheric activity associated with right/left hand choices; Donner et al., 2009; Gould et al., 2012; Wyart et al., 2012). A traditional view is that beta oscillations in decision-making reflect motor preparation only, i.e., a serial processing view where the effector-specific motor plan is the final step, after higher-order areas have reached a decision based on sensory input. However, as will be outlined below, accruing evidence points to a more direct involvement of beta oscillations in decision formation, which may or may not be linked to a specific motor plan.

### Dynamic accumulative updating

**Figure 3.**
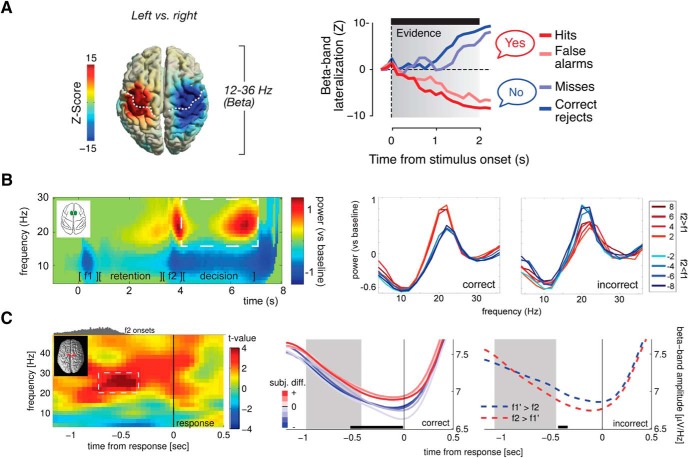
Content-specific beta activity during decision making. ***A***, Source reconstruction showing lateralized, effector-selective beta activity (left- vs right-hand response) before button press, in human subjects performing a visual motion detection task (left panel). Time courses of lateralized beta activity in motor cortex indicate accumulative updating of the motor plan as a decision evolves (right panel). Adapted with permission from Donner et al. (2009). Reprinted with permission from Elsevier. ***B***, Beta power modulation in monkey medial premotor cortex before motor response in vibrotactile discrimination task reflects binary decision outcome, also on error trials. Spectra on the right are averaged over the time window indicated by dashed box in the left panel, per stimulus class (sorted by f2-f1 difference), for correct and incorrect trials separately. Adapted with permission from Haegens et al. (2011b). ***C***, Remarkably similar observation as in ***B***, in human EEG recordings using the same paradigm. Adapted with permission from Herding et al. (2016). © 2016 by the Massachusetts Institute of Technology, published by the MIT Press.

Several recent studies suggest that lateralized beta activity during decision-making tasks may not only reflect terminal movement preparation, but a dynamic process of accumulatively updating a motor plan as a decision evolves (Donner et al., 2009; Gould et al., 2012; O'Connell et al., 2012; Wyart et al., 2012; Kubanek et al., 2013; Wyart et al., 2015). For instance, analyzing human MEG activity in a visual motion-detection task, Donner et al. (2009) reported a slowly evolving, gradual beta power lateralization in (pre)motor cortex that tracked the current state of evidence accumulation, as inferred from the temporal integral of gamma activity in motion-sensitive area MT (Fig. 3*A*). Similar observations were made in human EEG studies where participants integrated sequential samples of decision information over extended periods of time (Gould et al., 2012; Wyart et al., 2012; Kubanek et al., 2013; Wyart et al., 2015). In these studies, sensorimotor beta was found to reflect the integral of accumulated decision information in the form of a gradual response preparation signal, downstream to the encoding of sample-level decision information in parietal EEG signals (Gould et al., 2012; Wyart et al., 2012; Kubanek et al., 2013).

Together, these studies support a role for beta in dynamic updating of the decision outcome as mapped onto a motor response. However, a key point in the above studies is that they a priori operationalized beta activity as a response-related signal. More specifically, they used a fixed mapping between decision outcomes and motor responses (usually left/right hand action), and assessed sensorimotor beta activity in terms of lateralization indices, relying on the contra-lateralized nature of sensorimotor activity. Critically, this approach disregards choice-related activity that might occur independent of the associated left/right response mapping. In fact, when the fixed link between decision outcomes and left/right effectors is removed, the effects in sensorimotor beta lateralization typically disappear (O'Connell et al., 2012; Twomey et al., 2016).

### A content-specific decision signal?

Several recent studies go beyond this approach, and demonstrate a role for beta oscillations in perceptual decision tasks that appears independent of motor-response mapping (Haegens et al., 2011b; Herding et al., 2016; Stanley et al., 2016; Wimmer et al., 2016). Haegens et al. (2011b) used a variant of the somatosensory frequency discrimination task described earlier (Mountcastle et al., 1967; Romo and de Lafuente, 2013). As outlined above, during the retention period of such tasks, the first stimulus frequency (f1) is reflected parametrically in (pre)frontal areas, both in terms of single-cell firing rates (Romo et al., 1999; Barak et al., 2010; Vergara et al., 2016) and in upper beta activity (Spitzer et al., 2010). Notably, during the ensuing decision period (i.e., after f2 is presented), Haegens et al. (2011b) found that the level of beta power in monkey sensorimotor cortex (including somatosensory, premotor and motor areas) signaled the categorical outcome of the f1-f2 comparison (i.e., “f2 > f1” or “f1 < f2”). This effect proved independent of the absolute frequencies of f1 and f2 (or their exact numerical difference) and reflected the monkey’s categorical choices even on error trials (Fig. 3*B*). Importantly, this choice-related beta activity was not merely related to motor planning, as all choices were reported with the left hand, and the effect disappeared in a control condition in which the same motor response but no f1-f2 comparison was required. These findings complement previous reports on spike firing rate modulation in the same paradigm: during the comparison period, firing rates of sensorimotor and prefrontal cells gradually reflected a categorical response, corresponding to the decision outcome (Hernández et al., 2002; Romo et al., 2002; Hernández et al., 2010). Therein, similar to the “parametric” WM ensembles described earlier (Romo et al., 1999), decision-coding cells fall into two complementary classes, with one group of cells positively tuned (i.e., increasing its firing) to the f2 > f1 choice and another negatively tuned (Hernández et al., 2002).

A beta-band effect replicating and extending the monkey findings by Haegens et al. (2011b) was recently observed in human EEG recordings (Herding et al., 2016). Here too, during vibrotactile frequency discrimination, the level of nonlateralized beta power in premotor areas was modulated according to participants’ decision outcomes (f2 > f1 or f2 < f1) in a categorical fashion (Fig. 3*C*). Again, this effect reflected subjective choices, including errors, as inferred from Bayesian modeling of f1-f2 choice behavior. Furthermore, capitalizing on a larger subject sample, this effect was found to be invariant across motor response mappings: even when the response scheme (index or middle finger of the right hand) was flipped (across participants), the beta modulations remained unchanged (Herding et al., 2016). In a follow-up study, virtually identical beta patterns were observed when saccades rather than button presses were used to communicate the decision. Now decision-selective beta activity was localized to more lateral premotor areas (including the frontal eye fields), suggesting a degree of effector specificity in terms of areas involved, but with a consistent role for beta (Herding et al., 2017).

Combined, these studies suggest that in scalar comparison tasks, sensorimotor beta oscillations can reflect the categorical, potentially abstract content of a decision, even independent of a concrete motor plan. One possibility is that such nonlateralized, content-dependent beta activity relates to the endogenous activation of categorical, conceptual information before local translation into an effector-specific response. This interpretation is in line with a recent study recording LFPs in lateral PFC of macaques performing a visual categorization task, which reported different patterns of beta coherence for different categories of morphed stimuli (ranging for instance from cat to dog), “as if low-beta coherence was helping to form the neural ensembles that represented the categories” (Stanley et al., 2016). In further support of this view, when macaques had to judge random-dot motion stimuli, beta activity in lateral PFC signaled the categorical decision outcome (here, “same” or “different”), with beta modulation in different recording sites corresponding to the different outcomes (Wimmer et al., 2016). As for the somatosensory studies discussed above, this observation complemented findings from single unit spike recordings in the same paradigm (Hussar and Pasternak, 2012): different cells increased their firing rate either for same or for different choices. Thus, there appear to be parallels between modulations of local beta activity and single unit firing rates, in that both signal the emergence of a categorical decision outcome.

### Decision circuits

The role of beta oscillations in decision making might be extended to include long-range interactions, again in line with WM findings discussed earlier. The decision effects observed by Haegens et al. (2011b), for instance, included a distributed network of somatosensory and (pre)motor areas. More direct support for a role in network-level processing comes from a reach-planning study, demonstrating higher beta-band spike-field coherence (SFC; the synchronization of spikes to oscillatory phase) between premotor cortex and the parietal reach region when monkeys were freely making choices as compared to instructed choices (Pesaran et al., 2008). The authors proposed that here, beta coherence reflected a decision circuit between frontal and parietal cortex, which was more activated under free choice conditions. Similarly, beta-band SFC in posterior parietal cortex reflected decisions in a reward-guided choice task (Hawellek et al., 2016). In this study, information about movement choice in firing rates was quantified and related to the phase of beta and gamma oscillations. While for gamma, peak firing rate and maximum information content coincided, for beta the highest spike count preceded maximum information. These differences in temporal alignment were linked to the idea that gamma reflects local, bottom-up processing, while beta links distributed ensembles for computations on a larger scale. Further evidence for beta facilitating long-range communication was obtained in a recent auditory perceptual decision-making study, in which large-scale network dynamics in the beta-band predicted decision speed (Alavash et al., 2017).

To summarize, a growing body of evidence suggests that content-specific beta oscillations can signal the endogenous activation of a categorical decision outcome before translation into a concrete motor response. Several studies show that such content-specific decision activity in the beta-band can be observed beyond sensorimotor regions, both within and between distributed cortical areas.

## A role for beta oscillations in endogenous content (re)activation

**Figure 4.**
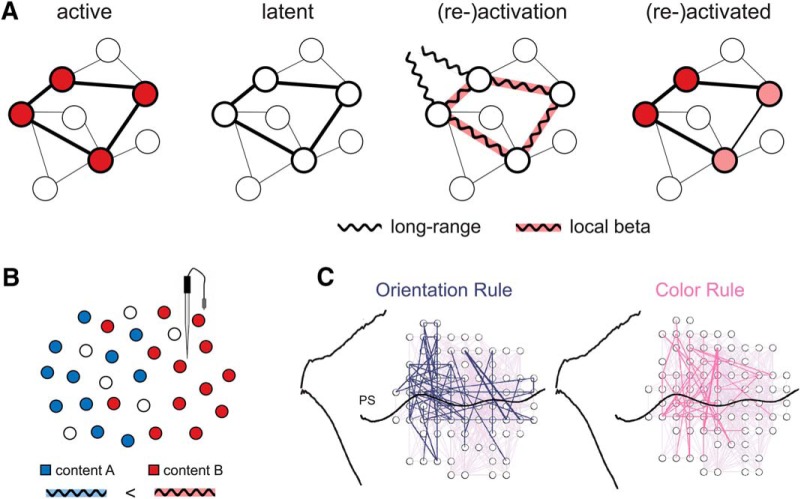
A framework for content-specific beta activity. ***A***, Content-specific beta-synchronization as endogenously driven transition from latent to active cortical representation. Left, Active cortical representations (e.g., of currently perceived, task-relevant information) are characterized by spiking activity (symbolized in red) in content-specific neuronal ensembles. Second from left, In the absence of perceptual input and/or attentional prioritization, information can be retained in latent memory representations, without spiking activity in the content-specific ensemble, e.g., in patterns of synaptic weights. Second from right, Endogenously driven (re)activation of a content-coding ensemble is characterized by a brief period of beta-synchronization, involving both local and long-range (top-down) interactions (see text for details). Right, (Re)activated content representations may again be characterized by spiking ensemble activity, similar (but not necessarily exactly identical) to representations of just perceived information (compare with left). ***B***, Local beta activity appears content specific when population-level recordings register the synchronization of individual subpopulations (symbolized in blue and red) with differential sensitivity (e.g., red > blue, by spatial proximity to recording site). ***C***, Transient network-level beta coherence in monkey PFC during application of different task rules (Fig. 2*F*, dashed rectangle). Adapted with permission from Buschman et al. (2012). Reprinted with permission from Elsevier.

In the previous sections, we have discussed research in the domains of WM and decision making, showing that beta activity can be modulated in a content-specific manner. Here, we outline a framework for beta oscillations in endogenous (re)activation of cortical content representations (Fig. 4*A*). We presume that active cortical representations of task-relevant information are reflected in the (spiking) activity of content-specific neuronal ensembles (Fig. 4*A*, first panel). We further assume that in the absence of stimulation or endogenous prioritization, representations of task-related information can persist without sustained ensemble spiking, for instance, in patterns of synaptic weights (Jonides et al., 2008). Such dormant, or latent memory representations (Fig. 4*A*, second panel) may for instance be characterized by short-term synaptic facilitation (Mongillo et al., 2008; Stokes, 2015) for just presented stimuli, and/or by long-term synaptic potentiation (Hebb, 1949) for overlearned (e.g., abstract/categorical) contents. A general assumption in this framework is that latent memory information can be endogenously restored into an active (i.e., spiking) cortical representation (Fig. 4*A*, last panel), for instance by top-down attentional prioritization (Warden and Miller, 2007; Jonides et al., 2008; Jacob and Nieder, 2014; Watanabe and Funahashi, 2014; Sprague et al., 2016). The mechanisms by which such endogenous (re)activation might occur, however, have thus far remained unclear. Here, based on the accumulating evidence reviewed above, we propose that this role is filled by content-specific beta-band activity. More specifically, we suggest that episodes of content-specific beta-synchronization support the endogenous transition from latent to active cortical representations (Fig. 4*A*, third panel), in the service of current task demands.

Oscillatory synchronization is associated with fluctuations in local network excitability (Bishop, 1932), and faster rhythms (>15 Hz) in particular are proposed to support flexible information routing by providing windows of efficient inter-areal communication (Fries, 2015; Palmigiano et al., 2017). Oscillations in the beta-band seem particularly well suited to fill these roles during endogenously driven information processing, given (1) their association with top-down processing (Engel and Fries, 2010; Wang, 2010) and (2) long-range communication (Kopell et al., 2000; Varela et al., 2001; Sherman et al., 2016), (3) their burst-like temporal dynamics (Jones, 2016), (4) their presumed role in the flexible formation and manipulation of functional cell assemblies (Roopun et al., 2008; Kopell et al., 2011), and (5) their capacity to modulate impact of neuronal firing (Buzsáki and Draguhn, 2004; Wang, 2010). While several of these characteristics may also apply to other rhythms, the combination of all these aspects appears unique to beta.

Mechanistically, the association of endogenously driven ensemble activation with beta oscillations is in line with models of beta generation that rely on integration of concurrent inputs along the proximal and distal locations of the apical dendrites of pyramidal cells (Jones et al., 2009; Sherman et al., 2016). This integration includes both feedforward (lemniscal thalamic) input via the granular layer, and, critically, feedback (higher-order cortical and/or nonlemniscal thalamic) drives to the supragranular layers (essential for beta emergence in the model), permitting top-down driven synchronization of a cell assembly, mitigated via cortico-(thalamo)cortical drives. Furthermore, based on models that rely on beta-rate spiking-interactions within local cortical circuits (Kopell et al., 2011), it has been argued that beta-synchronized ensembles are less susceptible to competition (unlike PING/gamma networks). In the present context, this property of beta might permit endogenous content activation to operate in a “protected” oscillatory regime that is relatively robust to distractor interference, e.g., from concurrent sensory inputs.

We may further speculate that beta is an ideal “transit” band for endogenously driven (re)activation, bridging the frequency space between alpha, which is commonly associated with top-down inhibition (Klimesch et al., 2007; Haegens et al., 2011a), and gamma, which is positively linked to population spiking (Whittingstall and Logothetis, 2009). Relatedly, previous modeling work (Lundqvist et al., 2010; Lundqvist et al., 2011) has characterized (WM-) reactivation as a transition from a low-frequency (alpha/beta) oscillatory regime (associated with a noncoding ground or “default” state) to a higher frequency/gamma regime (associated with active stimulus coding), similar to our conceptualization of latent and (re)activated representations (Fig. 4*A*, second and fourth panels). Superficially, the association of beta with a default state (Lundqvist et al., 2016; see also Engel and Fries, 2010) appears inconsistent with a role in content (re)activation. However, the two perspectives can be reconciled when considering that content specificity (in terms of experiment-related information; which might be dissociable from less specific, ongoing beta rhythmicity, see Future perspectives below) emerges only during the critical transition between representational states (Fig. 4*A*, third panel).

Our proposal captures various hallmarks of content-specific beta activity in the domains of WM and decision making. First, under this framework, content-specific beta episodes are expected to be relatively short lived (see also Jones, 2016; Sherman et al., 2016), since they would reflect neither latent nor active representations per se, but only a (presumably brief) transition period between the two (Fig. 4*A*). Consistently, content-specific beta modulations in WM tasks are typically observed in circumscribed time windows, in which participants should bring back past information into the focus of attention (Spitzer and Blankenburg, 2011; Spitzer et al., 2014b; Wimmer et al., 2016). Similarly, beta-oscillatory representations of task rules in monkey PFC (Fig. 4*C*) were short lived (Fig. 2*F*) and appeared only while a stimulus was to be evaluated according to the current rule (Buschman et al., 2012). Notably, in the Buschman et al., study, rules were only switched between blocks of trials, likely leading to a (latent) memory of the current rule that persisted across trials. A representation of such memory in beta-synchrony was indeed absent throughout large portions of the trial, and emerged only shortly before the to-be-evaluated stimulus, as if the current rule was endogenously (re)activated for task-oriented processing. Before and after this brief episode, prefrontal firing rates, but not beta-synchrony, encoded just-presented cues, consistent with our differentiation of purely endogenous (re)activation processes in the beta-band from active neuronal representations per se (Fig. 4*A*).

In a similar vein, the proposed framework can explain findings of content-specific beta activity during categorization and decision making, in dissociation from traditional indices of motor preparation. In categorization tasks, subjects are asked to select one of two (or more) internalized prototype concepts, which in our framework entails the endogenous activation of a stored content representation. Indeed, category-selective beta-synchronization during stimulus categorization was found only after extensive category learning (Antzoulatos and Miller, 2014, 2016), corroborating the idea that beta is especially involved in *re*activating cortical representations. Likewise, modulations of beta activity according to categorical decision outcomes, such as in the vibrotactile frequency comparison tasks described earlier, can be understood in terms of endogenously activating an abstract concept representation, e.g., “higher” (f2 > f1) or lower (“f2 < f1”). Indeed, on any given trial in the above tasks, the concepts or categories in question may coexist in form of latent representations, one of which will be activated at the time of choice, as reflected in content-specific beta activity.

Our framework is further consistent with a nontrivial relationship between beta oscillations and spiking activity (Whittingstall and Logothetis, 2009; Rule et al., 2017). Conceiving of content-specific beta activity as a transition period (Fig. 4*A*), temporal correlations with spike firing can be weakly negative or positive, depending on how strongly beta episodes overlap in time with (still) dormant or (already) activated representations. Furthermore, rather than in- or decreases of net firing rates in a given area, we assume a (content-specific) distribution of neuronal firing within and/or between functional ensembles. This idea is in line with the spatio-temporal coincidence of local beta modulations with a shifting of firing rates between oppositely tuned cell populations (Romo et al., 1999; Hernández et al., 2002; Barak et al., 2010; Spitzer et al., 2010; Haegens et al., 2011b; Hussar and Pasternak, 2012; Wimmer et al., 2016). In these contexts, beta activity may appear content specific to the extent that population-level recordings (such as M/EEG or LFP) register the oscillatory signatures of individual subpopulations with different sensitivity (Fig. 4*B*). As a corollary of this view, the sign of content-dependent beta modulations (e.g., whether local beta activity in- or decreases for a given content) might be noninformative and dependent on the particular recording setting. However, the precise relation between beta oscillations and spiking ensemble activity remains speculative and awaits further investigation.

Based on the available findings across primate species, endogenous content (re)activation can include modulations of beta activity both locally and in terms of long-range synchronization between distant regions (Fig. 1*B*). Modulations of local beta power have mostly been observed for low-dimensional information, such as scalar stimulus attributes (Spitzer et al., 2010; Haegens et al., 2011b; Wimmer et al., 2016). Higher-dimensional contents, such as object identity or task rules, have been associated with sophisticated patterns of beta-synchronization between multiple recording sites, potentially reflecting the activation of more distributed cortical representations (Buschman et al., 2012; Salazar et al., 2012; Antzoulatos and Miller, 2016). In all of these cases, beta seems to provide a flexible scaffolding that sets up functional neuronal ensembles through temporary synchronization of content-coding cell populations. The demand for flexibility in ensemble formation may be particularly high in regions with “mixed selectivity” cells (Rigotti et al., 2013), such as the prefrontal and parietal cortices, where single neurons respond to a multiplicity of task variables (for review, see Fusi et al., 2016). It might be especially in communication within and with these regions that frequency-specific synchronization finesses the active representation of internally stored information alongside current input, in potentially overlapping functional networks.

### Future perspectives

An open question remains whether transient content specificity of beta emerges from a modification of ongoing beta rhythmicity (cf. Engel and Fries, 2010; Lundqvist et al., 2016), or whether the two reflect functionally dissociable phenomena in overlapping frequency ranges. It is possible that the beta-band encompasses several rhythms, including a potentially “inhibitory” rhythm that is functionally more similar to alpha and which seems especially prevalent in somatomotor context (for review, see Kilavik et al., 2013). Indeed, the possibility that beta is not a unitary phenomenon but covers several roles may help to reconcile seemingly disparate observations, such as WM-load-related beta-power increases in some studies (Deiber et al., 2007; Kornblith et al., 2016), but decreases in others (Siegel et al., 2009; Lundqvist et al., 2011). Relatedly, several authors divide the beta-band into a lower (<20 Hz) and a higher (>20 Hz) subrange (Roopun et al., 2006; Kopell et al., 2011), with potentially distinct functional roles (see Introduction). In the literature reviewed here, however, we found only a weak, if any, tendency for content-specific effects (Fig. 1*A*, right) to occur in a higher beta frequency than overall, task-related modulations (Fig. 1*A*, left), with considerable variability across experiments, leaving the question of potentially distinct beta rhythms (and the determinants of their precise frequencies across cortical areas) to future targeted study.

A more general open question is the very nature of latent representations that are amenable to beta-mediated reactivation. As one possibility, content-specific beta activity might reflect a direct drive to reactivate activity-silent (e.g., synaptic) representations, as schematically illustrated in Figure 4*A*. In an alternative scenario, dormant memory representations are kept “silent” by actively inhibitory mechanisms, for instance, by content-matching “inhibitory engrams” (Ramaswami, 2014; Barron et al., 2017). Under this view, cortical reactivation may result from a release from inhibition, by suppression of inhibitory engrams, a scenario in which beta-mediated reactivation might indeed operate via inhibitory processes (“inhibition of inhibition”; Pfeffer et al., 2013). A related issue is the extent to which beta-mediated reactivation relies on the contents or concepts in question being familiar and consolidated in long(er)-term memory (which we assumed to be the case in most of the above reviewed studies). It remains to be shown empirically whether content-specific synchronization plays a role also in reactivating representations of entirely novel, just encountered information, a silent memory of which might persist only in transient patterns of short-term synaptic plasticity (Mongillo et al., 2008; Stokes, 2015).

Lastly, a key question for future work is how burst-like, transient beta events are temporally organized. One possibility is that temporal context is provided by other (lower) frequency rhythms that modulate beta via cross-frequency interactions. For instance, δ oscillations (1–3 Hz) are thought to tap into the temporal structure of behaviorally relevant events (reviewed in Merchant et al., 2015; cf. Lakatos et al., 2008; Schroeder and Lakatos, 2009), with faster oscillations “nested” in these slower rhythms. Such interactions might manifest in phase-amplitude coupling, where the phase of δ provides “windows-of-opportunity” for beta to burst. Indeed, there are indications that beta power can be modulated by δ oscillations in the context of WM (Siegel et al., 2009) and temporal prediction (Arnal et al., 2015; Herrmann et al., 2016). Such temporal structuring could be implemented via corticothalamocortical, and/or cortico-basal ganglia loops (cf. Merchant et al., 2015). For example, beta could be timed by bursting thalamic inputs (cf. Sherman et al., 2016), which in turn could be gated via the basal ganglia. Albeit speculative, these ideas are in line with studies showing that beta oscillations in the basal ganglia are associated with interval timing (Bartolo et al., 2014), providing promising avenues for future research.

## Conclusion

To summarize, we propose that content-specific beta-synchronization provides a mechanism for the formation of functional neuronal ensembles during endogenous (re)activation of cortical representations. This framework is in line with the emerging view that beta facilitates network-level communication (Kopell et al., 2000; Varela et al., 2001; Siegel et al., 2011) and specifically endogenous, top-down driven interactions (Engel and Fries, 2010; Wang, 2010; Arnal and Giraud, 2012; Bastos et al., 2012; Sherman et al., 2016). However, beyond a static role in maintaining the status quo (cf. Engel and Fries, 2010), we characterize content-specific beta-synchronization as a dynamic and highly flexible mechanism, one that can “wake up” (see also Fries, 2015), rather than merely preserve, an endogenous cognitive set. This proposal accommodates accumulating findings in animals and humans and outlines a functional role for beta that may fit its “burst-like” temporal characteristics (Jones, 2016). An intriguing question for future research is whether and how the beta-band dynamics discussed here interact with sensorimotor rhythms when (re)activated content representations are translated into concrete action plans.

## Synthesis

Reviewing Editor: Bradley Postle, University of Wisconsin Decisions are customarily a result of the Reviewing Editor and the peer reviewers coming together and discussing their recommendations until a consensus is reached. When revisions are invited, a fact-based synthesis statement explaining their decision and outlining what is needed to prepare a revision will be listed below. The following reviewer(s) agreed to reveal their identity: Tim Buschman, Rodrigo Salazar

Reviewer #1

Beyond the status quo: a role for beta oscillations in endogenous content (re-)activation

Historically, beta-band oscillations (~15-30 Hz) have been thought to be involved in motor planning; specifically, inhibiting the production of motor actions. However, recent work has begun to emphasize the role of beta-band oscillations in cognition, playing a role in attention, working memory, perception, and decision making. In particular, the pattern of beta-band oscillations across the brain often reflect the state of the brain (e.g. reflecting the content of working memory). This manuscript nicely reviews the recent literature on beta-band oscillations; consolidating it into a new model of beta oscillations that suggests they act to temporarily reactivate neural representations. This is consistent with a role for beta oscillations in ‘top-down’ control, and can explain disparate findings in working memory and decision making. Overall, the manuscript is timely, well written, clearly describes recent work and synthesizes it into a cohesive theory. I only have a few comments:

1. The authors provide a nice review of current theoretical models that generate beta oscillations. However, this is never woven back into the final theory. One obvious point of contact is Nancy Kopell's models that suggest beta oscillations are ideal at forming cell assemblies (e.g. Kopell et al, 2011 and others). However, the author also nicely reviews literature associating beta-band oscillations with cortico-ganglia interactions and with specific cortical layer interactions. A connection between these models and how/why they play a re-activation role in cortex would strengthen the current model.

2. My understanding of the Lundqvist et al model was that beta-band oscillations play a role in content-less reactivation and that higher-frequency, gamma-band oscillations are involved in reactivating memories. This seems to strongly differ from the beta-band content-specific activation suggested here. Obviously, as reviewed here, experimental work does support the content-specific model, however, a deeper comparison between the two models might help differentiate the theories. For example, the Lundqvist et al models suggest the gamma-band activation would be ideal for the short-term plasticity mechanism - how would the current model account for such arguments?

3. Related to comment #1 and #2, but more generally, it isn't clear why beta-band oscillations would be useful for (re-)activating cortical ensemble representations. Do the author(s) have a hypothesis on why beta-band specifically is useful for such a mechanism?

4. One related finding is that the overall beta-band power seems to decrease with the number of items held in working memory (Siegel et al, 2009; although this effect seems weaker in Lundqvist et al, 2016). Does this argue against the proposed role for beta-band oscillations in re-activating items in memory? One would expect that, if overall beta power reflects the burst rate (or duration) of beta-band bursts, then more items in memory should correlate with an increase in beta-band power?

Reviewer #2

The manuscripts titled “Beyond the status quo: a role for beta oscillations in endogenous content (re-) activation” present a novel theoretical framework for the function of beta oscillations in primate brains. The novelty resides in their proposed role in transitioning latent neuronal ensembles into “active” representations by influencing the spiking activity of the participating neurons. In this line of thinking, they highlight the need to clarify the relationship between beta oscillations and firing activity. Overall, they cite to up-to-date referenced literature, review several hypotheses about the generation of beta oscillations and finally propose a highly simplified mechanism to achieve the re-activation of neuronal ensembles. The style and the conceptual organization of the manuscript guide the reader nicely through the different concepts and evidence.

I only have a few suggestions to improve and further complement the current state of the manuscript:

1) The presentation of results about other frequency bands (theta, alpha and gamma) is currently embedded with findings about the beta frequency band. I believe that this approach is distracting the reader from the main points. I would rather make a specific paragraph about the relationship between beta and the other frequency bands. If the authors decide to make this effort, I would highly suggest highlighting the lack of knowledge on cross-frequency modulations of beta oscillations and the other frequency bands.

2) The resistance to distractors is a classical approach to dissociate “latent” from “active” representations. It would be a nice add-on to the review to summarize current knowledge and future research directions in this topic.

3) I am not fond of the simplicity of the neuronal mechanisms proposed in the current manuscript. I believe that the authors should go into more details at many levels. For example, the involvement of synaptic plasticity is shortly mentioned in the text but does not at all appear in their framework (Figure 4A). I believe that most experts in the field expect a critical role of synaptic plasticity in these processes. Given the little amount of experimental data, the authors have the opportunity to speculate and drive future experiments in this direction. Another issue worthwhile addressing is why is the beta band particularly well suited instead of other frequency bands. The current framework would gain a strong interest if some physiological properties of beta oscillations were provided to explain their role.

4) The authors emphasize the transient nature of beta bursts but their framework highlights the endogenous predictive nature of beta oscillations about the timing to (re)-activate relevant neuronal representations. Can the authors speculate about the mechanisms involved in timing behavioral events when beta oscillations are transient? Suggesting such a mechanism would inspire future experiments.

5) The distinction between low and high beta oscillations and their potential different roles is only shortly addressed. The review would be more complete if the authors summarize current knowledge in this topic.
